# Remediation of Surfactants Used by VUV/O_3_ Techniques: Degradation Efficiency, Pathway and Toxicological Analysis

**DOI:** 10.3390/molecules28083312

**Published:** 2023-04-08

**Authors:** Hang Li, Yanling Yang, Xing Li, Habib Ullah

**Affiliations:** 1 College of Architecture & Civil Engineering, Faculty of Urban Construction, Beijing University of Technology, Beijing 100124, China; 17810299661@163.com (H.L.);; 2 Department of Environmental Science, Zhejiang University, Hangzhou 310058, China

**Keywords:** ozone, surfactants, toxicological analysis, vacuum ultraviolet

## Abstract

Surfactants are increasingly used in systems that come into contact with the human body, such as food, pharmaceuticals, cosmetics and personal hygiene products. Increasing attention is being devoted to the toxic effects of surfactants in various human contact formulations, as well as the removal of residual surfactants. In the presence of ozone (O_3_), anion surfactants—a characteristic micro-pollutant—such as sodium dodecylbenzene sulfonate (SDBS) in greywater, can be removed using radical advanced oxidation. Herein, we report a systematic study of the SDBS degradation effect of O_3_ activated by vacuum ultraviolet (VUV) irradiation and the influence of water composition on VUV/O_3_, and determined the contribution of radical species. We show a synergistic effect of VUV and O_3_, while VUV/O_3_ reached a higher mineralization (50.37%) than that of VUV (10.63%) and O_3_ (29.60%) alone. The main reactive radicals of VUV/O_3_ were HO•. VUV/O_3_ had an optimal pH of 9. The addition of SO_4_^2−^ had almost no effect on the degradation of SDBS by VUV/O_3_, Cl^−^ and HCO_3_^−^ slightly reduced the reaction rate, and NO_3_^−^ had a significant inhibition on the degradation. In total, SDBS had three isomers, with which the three degradation pathways were very comparable. Compared with SDBS, the toxicity and harmfulness of the degradation by-products of the VUV/O_3_ process decreased. Additionally, VUV/O_3_ could degrade synthetic anion surfactants from laundry greywater effectively. Overall, the results show the potential of VUV/O_3_ in safeguarding humans from residual surfactant hazards.

## 1. Introduction

The biological changes caused by surfactants and their metabolites in the body, i.e., the possible toxic side effects on the body, include acute toxicity, subacute toxicity, chronic toxicity, reproductive effects, embryotoxicity, teratogenicity, mutagenicity, carcinogenicity, allergenicity, hemolysis, etc. [1]. Surfactants are used as various processing aids or synergists in the food and pharmaceutical industries. This increases the exposure of surfactants to the human digestive tract and blood system. Hence, strict requirements are imposed on the oral toxicity, hemolysis, heredity, carcinogenicity and teratogenicity of surfactants [2]. When the concentration of surfactants is relatively high, it can adversely affect aquatic organisms [3]. For example, chronic and sublethal toxicity to aquatic animals occurs when the concentration of anionic surfactants exceeds 0.1 mg/L [4]. Surfactants also inhibit the growth, activity and photosynthetic capacity of aquatic algae [5]. Moreover, a number of synthetic surfactants and their decomposition products do present a potential risk to human health and the environment, and they tend to increase the problem of water treatment [1].

Surfactants are a class of chemicals that reduce the surface tension of liquid or liquid interfaces. They have hydrophobic tails and hydrophilic heads. Surfactants are usually found as monomers, but sometimes in more complex polymeric forms [6]. According to the charge of hydrophilic groups of surfactants, they are divided into anionic, cationic, and nonionic, semi-polar, and amphoteric. SDBS is a typical anionic surfactant, which has been extensively considered as a general pollutant in human activities [7]. Conventional biological methods, such as aerobic treatment or activated sludge tanks, can degrade surfactants efficiently, but are not suitable for the treatment of high concentrations of surfactants [8]. Physical and chemical treatments, such as adsorption, membrane separation, coagulation, electro-coagulation, electrochemical oxidation, microwave assisted degradation, and oxidizing treatments are applied for the removal of surfactants [9,10,11]. Among these technologies, advanced oxidation processes (AOPs) based on hydroxyl radicals (HO•) are the most effective method for SDBS removal, which is a key stage in the treatment of greywater [11,12,13]. Moreover, the AOPs prevent foam and pathogens in the subsequent greywater treatment process and increase the biochemical properties of the greywater [14]. AOPs rely on the generation of radical species to rapidly degrade and disinfect contaminants in water and are an alternative method for greywater reuse applications. These radical species readily react with pollutants containing electron-rich groups [14]. Further, they offer the advantage of degrading organic pollutants and removing pathogens rather than transferring them to a concentrated stage for treatment. Ozonation has been shown to be effective in treating greywater by effectively removing organic pollutants from greywater [15,16,17,18]. However, ozonation has difficulty in completely mineralizing organic matter, and has a low usage rate [19]. In addition, ozonation treatment of wastewater may also produce disinfection by-products [20]. To overcome these problems, researchers have proposed catalytic ozone oxidation to improve the yield of radicals and the removal efficiency of pollutants from water [19,21,22]. The combination of ozone and UV is considered to be an effective method to enhance the removal of organic pollutants by ozonation [23].

To enhance the oxidation efficiency of UV/O_3_, VUV (100–200 nm) is gradually becoming an alternative to UV because of its environmentally-friendly and efficient characteristics [24]. The VUV wavelengths used have good photochemical activity, with 185 nm photons having higher energy and usage rates than 254 nm photons to activate oxidants and so to produce strong oxidizing HO• [25]. At 185 nm, the majority of photons are captured by H_2_O, leading to homolytic cleavage and the production of powerful oxidizing hydroxyl radicals [26,27,28]. Compared with O_3_ treatment, VUV/O_3_ treatment significantly accelerated the utilization of O_3_ and the yield of HO•. VUV/O_3_ can decrease the acute toxicity of wastewater significantly and enhance the degradation of refractory organic matter in biological treatment [29]. Moreover, the VUV/O_3_ process of treating sodium n-butyl xanthate improved the COD removal by 30.4–41.6% compared to the O_3_ process, and O_3_ usage in the VUV/O_3_ process increased by 17.3–45.1% [30]. Compared to O_3_, VUV, UV/O_3_, and UV processes, the VUV/O_3_ process was effective in removing normal organic matters and performed best in DOC mineralization, UV_254_ reduction, and biodegradability (BDOC/DOC) enhancement [31]. These studies demonstrated the advantages of VUV/O_3_ in the oxidative degradation of organic contaminants in greywater. In addition, the VUV/O_3_ process is simple to use and occupies a compact footprint. However, the application of the VUV/O_3_ process to the removal of the characteristic pollutant SDBS in greywater has rarely been researched. Therefore, the effectiveness of VUV/O_3_ in eliminating SDBS, and the mechanism to enhance the oxidation effect of O_3_, need a more in-depth investigation.

The role of VUV/O_3_ in the oxidative degradation of SDBS and its mechanism were investigated. Electron paramagnetic resonance (EPR) and quenching tests were used to elucidate the mechanism of radical species action. The effects of the solution components on VUV/O_3_ were studied, involving changes of initial SDBS concentration, solution pH, as well as the co-existence of inorganic anions. The reaction sites and degradation pathways of SDBS during VUV/O_3_ were determined and the toxicological properties of the intermediates were evaluated. Finally, the applicability of VUV/O_3_ in the removal of anionic surfactants from real laundry greywater was verified. This study can help to more deeply understand the reaction mechanism of VUV/O_3_ and provide new concepts for remediation techniques for surfactants.

## 2. Results and Discussion

### 2.1. Degradation Rule of SDBS during Various Treatments

**Figure 1:**
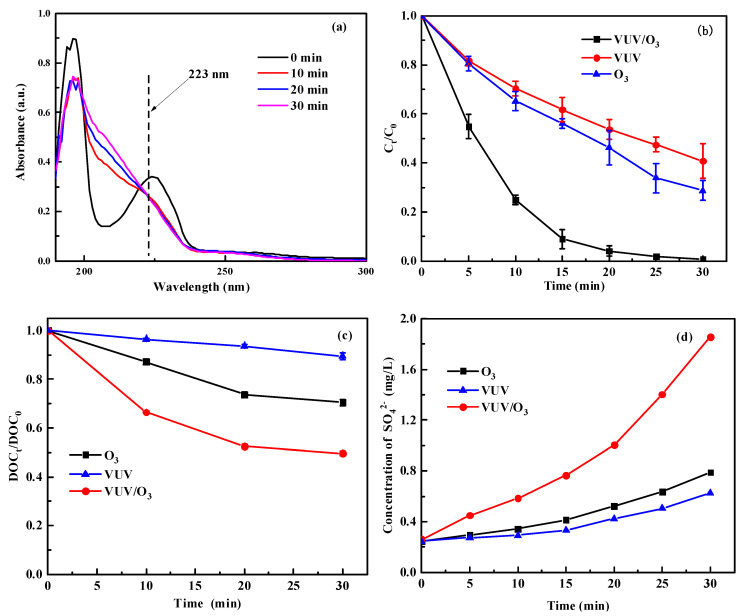
Rules of SDBS degradation by the VUV/O_3_ process: (**a**) UV-Vis spectra, (**b**) removal efficiency, (**c**) mineralization, and (**d**) SO_4_^2−^ concentration.

Figure 1 illustrates the procedure of SDBS degraded by O_3_ treatment, VUV treatment, and combined VUV/O_3_ treatment. During the 30 min of reaction, VUV/O_3_ was efficient to degrade SDBS at the 223 nm optical band as shown in Figure 1a. Based on the DFT calculation as shown in Figures S2–S4, 16(C)–21(C) on-ring bonds were the more vulnerable sites to radicals’ attack than the (C) on carbon chain. Interestingly, with the progress of VUV/O_3_ treatment, the absorbance peak widened, and a visible shoulder peak appeared, indicating the presence of intermediates. According to previous studies, SDBS can be effectively degraded by O_3_ itself as well as by generating free radicals, and is more efficient than other commonly used oxidants [11]. At the same time, O_3_ can be activated by VUV irradiation to increase the yield of radical species, which can enhance the degradation effect of surfactant [11]. Overall, it can be concluded that VUV/O_3_ was an effective method for degrading surfactants.

Figure 1b shows the removal efficiency of SDBS by different treatments. It can be observed that C_t_/C_0_ progressively reduced with time, demonstrating an effective removal of SDBS. The removal efficiency of SDBS by single VUV and single O_3_ were only 59.21% and 71.27% when the time 30 min, respectively. The VUV/O_3_ had a better effect by 99.35% for SDBS removal. It can be found that VUV/O_3_ can promote the removal efficiency of SDBS by O_3_ due to its higher radical species yield.

Figure 1c depicts the mineralization of SDBS by the above three treatments during a 30 min reaction time. It can be seen that the DOC_t_/DOC_0_ gradually decreased with time, indicating a gradual increase in the mineralization of SDBS. The effect of single VUV on the removal of DOC from SDBS solution was not obvious, and the removal efficiency of DOC by single O_3_ was only 29.60%. The coupling of VUV and O_3_ had a high mineralization capacity (50.37%) for SDBS and showed a synergistic effect. This is due to the fact that O_3_ is able to generate radical species via other pathways as the VUV was activated, at which point O_3_ utilization increased significantly [29]. As the removal of SDBS by VUV/O_3_ reached 99.35% during 30 min, these results suggest that the generated intermediates make an important contribution to the determination of DOC. It is clear that VUV combining with O_3_ produced a synergistic effect and enhanced the mineralization capacity of O_3_.

Figure 1d shows the concentration of SO_4_^2−^ for the three treatments. The production of SO_4_^2−^ was significantly accelerated after 30 min of VUV/O_3_ treatment, but the effect of O_3_ and VUV treatments were not obvious and the final concentrations of SO_4_^2−^ were 1.856 mg/L, 0.788 mg/L and 0.628 mg/L, respectively. According to DFT calculation in Tables S1–S3, 1(S) is most susceptible to attack by radicals to produce SO_4_^2−^, and their presence implies that advanced oxidation was effective to cleavage the S-O bond. Higher energy photons enhanced the activated efficiency of O_3_ and led to the acceleration of the required electron transfer, which may account for the increased rate of SO_4_^2−^ production [32]. Overall, this may imply the oxidation capacities of VUV alone and O_3_ alone were enhanced by the coupling.

### 2.2. Types and Roles of Radicals Existing in VUV/O_3_ Process

**Figure 2:**
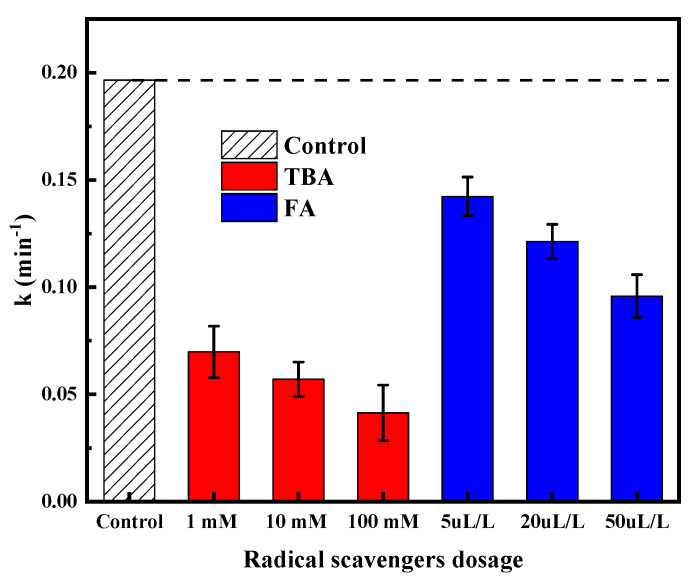
Scavenging tests during VUV/O_3_ process.

In order to obtain a quantitative evaluation of the role of various radicals on the SDBS degradation, the relevant reaction rate constants were calculated from the gradient dose of scavengers. The results of the research are displayed in Figure 2. The inhibitory degrees of SDBS degradation of two used scavengers were correlated with the oxidative effect of the relative radicals. As the TBA concentration increased from 1 to 100 mM, the degradation efficiency of SDBS by VUV/O_3_ decreased by 64.45–78.92%; for FA there was less of a concentration effect but an overall stronger inhibition: 27.58–51.24% for 5 μL/L–50 μL/L FA, respectively. Since the inhibition of the reaction was evident by the injection of low concentrations of TBA, it indicated that HO• was the main active substance in the degradation of SDBS by VUV/O_3_. Notably, the inhibition of the FA increased markedly with the increase of FA concentration. However, FA can preferentially absorb 185 nm photons over other components (H_2_O, O_3_, SDBS), so it can be inferred that O_3_ could also degrade SDBS, but not as effectively as HO• [31]. The results showed that both scavengers inhibited the degradation of SDBS during VUV/O_3_, and the degree of inhibition increased significantly with the increase of scavenger concentrations. It is thus clear that HO• played a more important role than UV photons and O_3_ in the degradation of SDBS during the VUV/O_3_ process.

**Figure 3:**
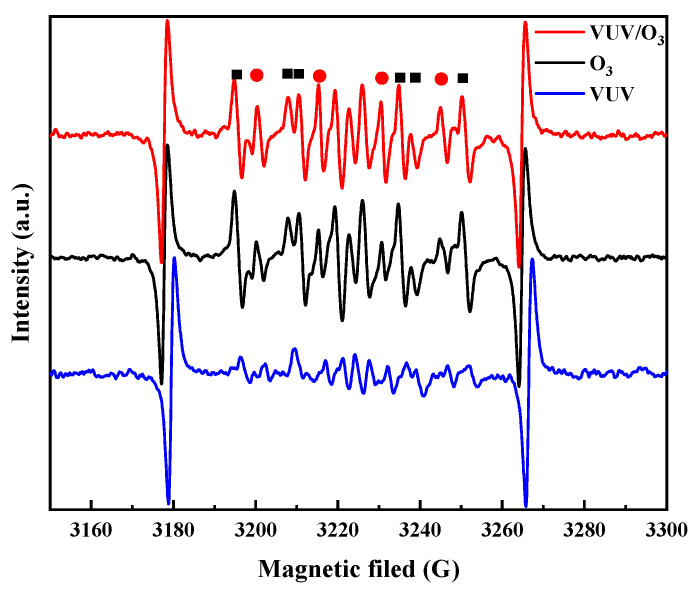
Radical species analysis at various process: HO• (red circles) and HOO• (black squares) formed in VUV/O_3_ process.

To further clarify the role of radicals, EPR spectra were performed to record. According to Figure 3, a 1:2:2:1 DMPO-OH characteristic peak spectrum could be recorded in O_3_ alone, and the intensity of such peaks was relatively diminished in VUV alone. Obviously, significant increase in the signal intensity of HO• by O_3_ under VUV activation [33]. This explains why the mineralization and concentrations of SO_4_^2−^ can be considerably increased during SDBS degradation by the VUV/O_3_ system. From the analysis of each EPR spectrum, it is clear that VUV led to a considerable activated efficiency of O_3_, producing more HO• as Equations (1)–(3). All these confirm that HO• was the main active substance in the VUV system and its effect was greater than that of O_3_.

H_2_O + hv_185nm_→ HO• + H• Φ_1_ = 0.33 (1)

H_2_O + hv_185nm_→ H^+^ +e_aq_^−^ + HO• Φ_2_ = 0.045 (2)

O_3_ + H_2_O + hv_254nm or 185nm_→ O_2_ + H_2_O_2_ + hv_254nm or 185nm_→ 2HO• (3)

### 2.3. Impacts of Varied Environmental Factors

#### 2.3.1. Impacts of Varied Initial O_3_ Concentrations on VUV/O_3_

**Figure 4:**
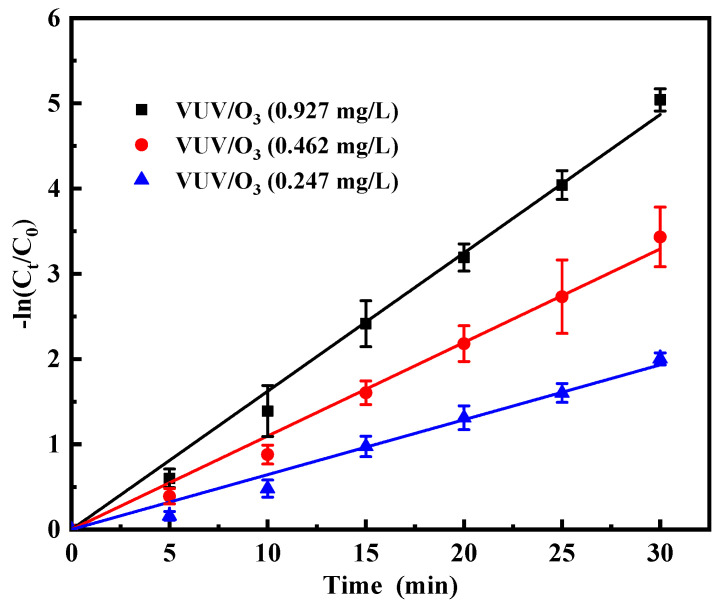
Impact of initial O_3_ concentration ([O_3_]_0_) on SDBS degradation by VUV/O_3_. Conditions: [O_3_]_0_ was changed from 0.247 to 0.927 mg/L.

As shown in Figure 4, the removal rate of SDBS gradually increased with increasing ozone concentration. The constant k (SDBS removal) considerably increased from 0.064 to 0.162 min^−1^ with O_3_ dosage ranging from 0.247 to 0.927 mg/L for the VUV/O_3_ process. According to the calculations, at ozone doses of 0.927 mg/L, the k was 1.5- and 2.5-fold larger than the k for 0.247 mg/L and 0.462 mg/L, respectively. Increasing the ratio of ozone concentration to surfactant concentration may increase the surfactant removal rate. This was mainly due to an increase in O_3_ concentration that increased the absorption efficiency of 185 nm and 254 nm photons, which led to an increase in the yield of radicals. Furthermore, ozone itself can be involved in SDBS degradation. Thus, an increase in ozone concentration can significantly increase the removal rate of SDBS. The result demonstrated that the decomposition of SDBS was highly effective during the VUV/O_3_ process, and that the removal of SDBS was facilitated by increasing the concentration of dissolved O_3_.

#### 2.3.2. Impacts of Varied Initial SDBS Concentrations on VUV/O_3_

**Figure 5:**
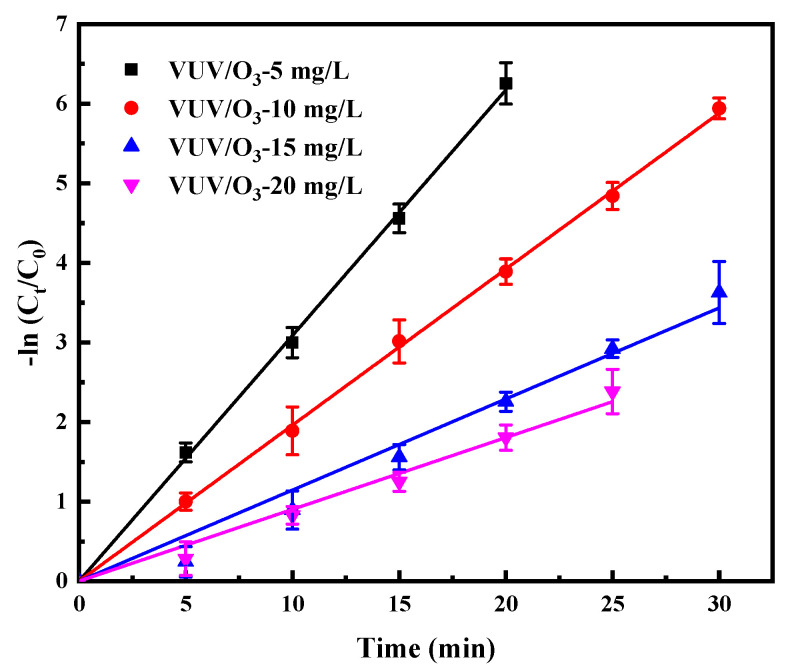
Impact of initial SDBS concentration ([SDBS]_0_) on SDBS degradation by VUV/O_3_. Conditions: [SDBS]_0_ was changed from 5 to 20 mg/L.

Figure 5 depicts the results predicted by a pseudo-first-order kinetic model of SDBS degradation by VUV/O_3_ when [SDBS]_0_ was varied in the range of 5 mg/L to 20 mg/L. A significant decrease in VUV/O_3_ oxidation efficiency was observed by increasing [SDBS]_0_, with the k for SDBS degradation decreasing from 0.309 to 0.090 min^−1^. This could be explained by the fact that the concentration of SDBS affected the absorbance of different components of the solution under 185 nm and 254 nm photons. Hence, the proportion of direct photolysis in the reaction was changed, as well as the proportion of O_3_ and H_2_O photolysis. Further, when the ozone concentration is constant, an increase in the concentration of SDBS leads to an increase in the priority of ozone reaction with SDBS. However, the redox potential of ozone is lower than that of HO•.

**Figure 6:**
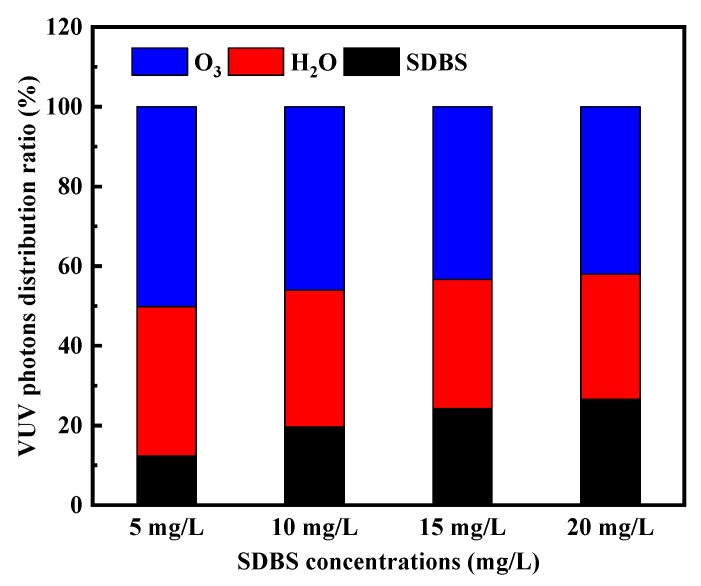
Impact of [SDBS]_0_ on the ratio of absorbance of various solution components to 185 nm photons.

As the concentrations were increased from 5 to 20 mg/L, the percentage absorbance of 185 nm photons by SDBS in competition with O_3_ and H_2_O increased from 12.31% at 5 mg/L to 19.58%, 24.27%, and 26.61% as the concentrations increased from 10 to 20 mg/L, as depicted in Figure 6 [34]. In theory, the higher the absorption ratio of O_3_ and H_2_O to 185 nm photons, the higher the activation efficiency of 185 nm photons. Since the concentration of O_3_ dissolved in solution remained constant, an increase in [SDBS]_0_ led to a decrease in the concentration of produced radicals, which would reduce the chance of effective collisions of radicals with contaminant molecules. Similarly, a previous study has also revealed that the removal of SDBS at lower rates by VUV/O_3_ for higher initial concentrations of contaminants [35].

The yield of radicals was constant for a fixed reaction time at the same ozone concentration, and the radical/reactant ratio decreased as the initial concentration of SDBS increased, leading to a decrease in the SDBS removal rate. Despite this, the degradation efficiency of SDBS was still very high even at high concentrations. The results show that VUV/O_3_ is a promising method for effective removal of anionic surfactants in greywater treatment [36].

#### 2.3.3. Performance of VUV/O_3_ in Varied Initial pH

**Figure 7:**
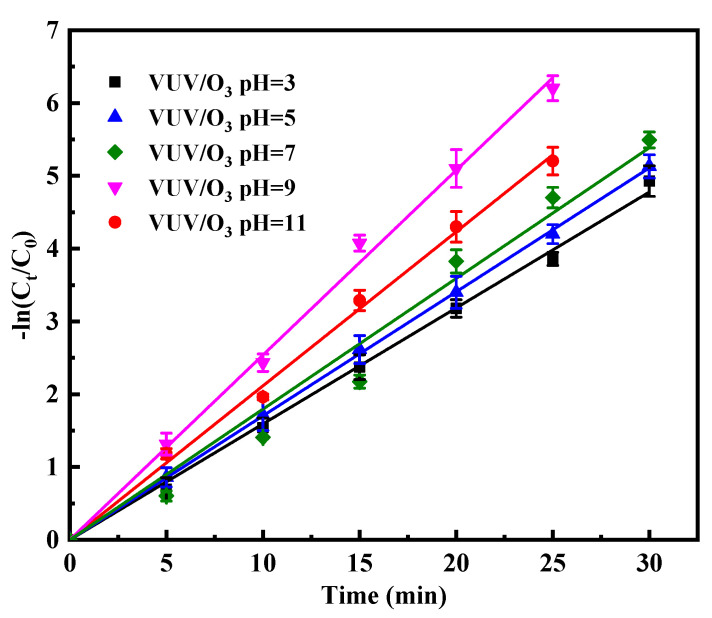
Impact of initial pH on the SDBS degradation during VUV/O_3_ treatment.

Figure 7 depicts the change in the degradation rate of SDBS degraded by VUV/O_3_ as the pH increases from 3 to 11. Solution pH is a major environmental variable, among others, that affects the degradation rate of advanced oxidation reactions, as it may determine the applicability of a process under practical treatment. The change in pH value resulted in the highest degradation rate of VUV/O_3_ process under weak alkaline conditions: when pH value is 9, the K value of VUV/O_3_ is 0.254 min^−1^. The rate of VUV/O_3_ oxidation reaction was higher in an alkaline environment. SDBS can exist as dodecylbenzene sulfonic acid under acidic conditions, and the undissociated SDBS is not as vulnerable to oxidative HO• as the anionic form [37]. Meanwhile, under the strong acidic condition, O_3_ oxidation dominates the degradation of SDBS and excess H_3_O^+^ might become a scavenger for HO•, which increased the consumption of reactive HO• (Equations (4) and (5)), thus inhibiting the degradation of SDBS [38]. In the VUV/O_3_ system, the higher the amount of HO^−^, the faster the decomposition of O_3_ to HO•, which is responsible for the increased degradation rate of SDBS in a high pH environment. HO• was a more reactive than O_3_, and alkaline conditions improved the increase of HO• yield, which facilitated the removal of SDBS [19,39]. The optimal pH was 9 rather than 11, indicating that HO• had a stronger oxidizing effect under weakly alkaline conditions and on ionized form of SDBS (DBS^−^) [40]. Previous studies have also observed comparable effects of pH upon the VUV/O_3_ process. Jiang et al. found that the optimal treatment condition by VUV/O_3_ was at pH of 9.0 [29], and Zhang et al. found that the removal rate of sodium n-butyl xanthate increased with increasing alkalinity of the solution in VUV/O_3_ process [30]. Experimental data verify that pH has a significant influence on the degradation of SDBS by VUV/O_3_, which is increased in strongly alkaline environments.

O_3_ + OH^−^ → HO• (4)

H^+^ + HO• + e^−^ → H_2_O (5)

#### 2.3.4. Implications of Typical Anions on SDBS Degradation

**Figure 8:**
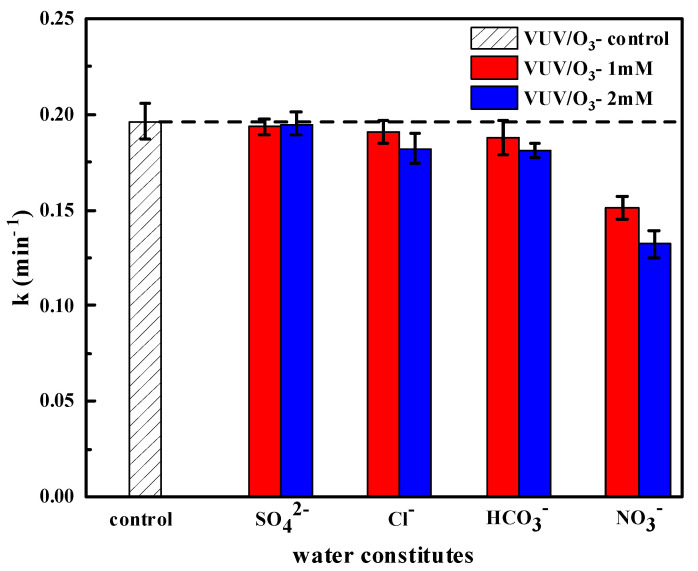
Impact of different inorganic anions (1–2 mM).

The implications of varied typical anions on the SDBS degradation during VUV/O_3_ process was examined, as shown in Figure 8. For the VUV/O_3_ process, the presence of SO_4_^2−^ hardly affected SDBS degradation; there was almost no decrease in the degradation rate when 1 mM and 2 mM SO_4_^2−^ were dosed. When 1 mM and 2 mM of Cl^−^ and CO_3_^−^ were present in the water, the degradation rate of SDBS decreased only slightly, with Cl^−^ causing a decrease of 2.75% and 7.25%, respectively, and CO_3_^−^ causing a decrease of 4.50% and 7.75%, respectively. Unlike these, there was a substantial decrease in the degradation rate of SDBS when 1 mM and 2 mM NO_3_^−^ were added, reaching 23.09% and 32.72%, respectively. The coexisting anions could trap the HO• radical species and convert them to second-order anionic radicals bearing relatively low redox potentials, according to chemical reaction formulae outlined as Equations (5)–(10) [41,42]. The SO_4_^2−^ could capture HO• and 185 nm photons to produce SO_4_^•−^ (Equations (5) and (6)), and to some extent, their redox potentials (HO•: 2.8 V, SO_4_^•−^: 2.5–3.1 V) are similar. The transient lifetime of SO_4_^•−^ is longer (HO•: 20 ns, SO_4_^•−^: about 30–40 μs) [41,43], so the combined reaction of multiple radicals does not influence the degradation rate of SDBS. A number of studies have found that sulfate radicals are similar in efficiency to hydroxyl radicals for the removal of certain micropollutants, and even higher for the removal of some pesticides [44,45]. As present Cl^−^, it can directly react with O_3_ to generate Cl_2_ or HOCl at low pH conditions [35], and they will undergo oxidative degradation reaction with SDBS. However, studies have reported that Cl• could react with H_2_O to form HO•, which might improve the degradation rate of SDBS to a certain degree [46]. These are the reasons for the diminished inhibitory degree of Cl^−^ on removal efficiency of SDBS during the VUV/O_3_ process. The reaction of HCO_3_^−^/CO_3_^2−^ with HO• to produce CO_3_^•−^ is the main radical in resolution at higher HCO_3_^−^ concentrations [47]. The redox potential of CO_3_^•−^ is significantly lower than that of HO•, which would decrease the effectiveness of SDBS removal. Since SDBS is easily degraded, CO_3_^•^ could also participate in the reaction, and the addition of HCO_3_^−^ might increase the solution pH, which enhanced the generation of HO•. The above reasons could alleviate the decrease of SDBS degradation rate by HCO_3_^−^. It was obtained that NO_3_^−^ had the greatest inhibition on SDBS degradation in the VUV/O_3_ process. NO_3_^−^ could absorb more 185 nm photons than any other because of its higher molar absorbance coefficient at 185 nm wavelength [41,48]. This led to a decrease in HO• yield and the production of lower oxidizing anionic radicals (NO_3_•), which inhibited the degradation of SDBS. Thus, these anions could also compete with H_2_O and O_3_ molecules to absorb 185 nm photons. In conclusion, the presented results showed that HO• can degrade SDBS more effectively than other radical species.

SO_4_^2−^ + HO• → SO_4_^•−^ + OH^−^ (6)

(7) $${{\mathrm{SO}}_{4}}^{2-}\;\overset{\mathrm{hv}\;<\;200\mathrm{nm}}{\to }\;{{\mathrm{SO}}_{4}}^{•-}+{e_{\mathrm{aq}}}^{-}$$

Cl^−^ +HO• → Cl• + OH^−^, *k* = 3.0 × 10^9^ M^−1^s^−1^ (8)

HCO_3_^−^ +HO• → CO_3_^•−^ + H_2_O, *k* = 8.6 × 10^6^ M^−1^s^−1^ (9)

NO_3_^−^ + HO• → NO_3_• + HO^−^ (10)

### 2.4. Proposed Degradation Pathways of SDBS

**Figure 9:**
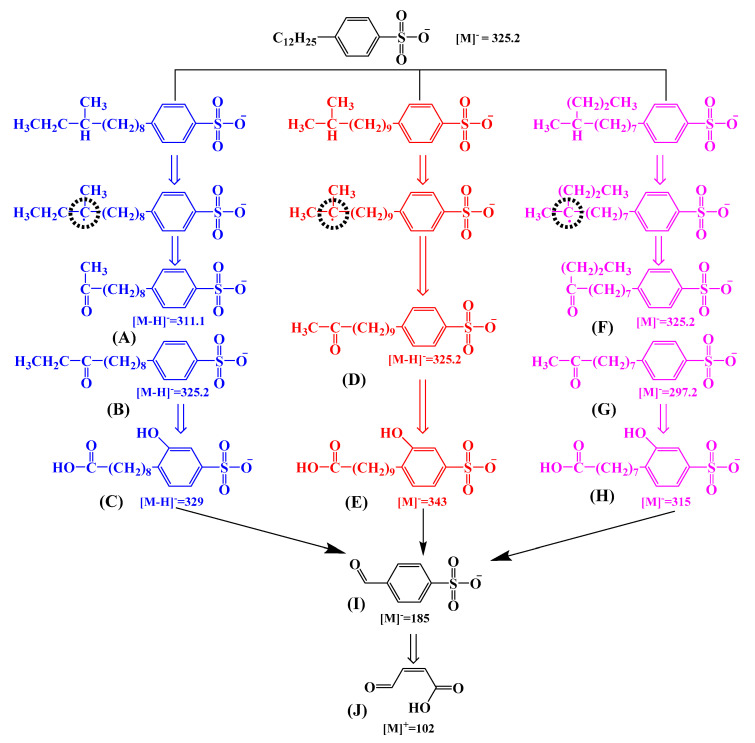
Proposed pathways of three SDBS isomers degradation by VUV/O_3_.

In order to derive a hypothetical pathway for the SDBS degradation during the VUV/O_3_ process, the formation of by-products was analyzed by high performance liquid chromatography/mass spectrometry (HPLC/MS) in ESI (−) and ESI (+) modes, and HOMO, LUMO, and Fukui indices of SDBS were calculated using DFT (Figures S2–S4 and Tables S1–S3). The obtained total ion chromatogram (TIC) and mass-to-charge ratio are presented in Figure S5. Ten by-products were deduced, and their structural formulae are listed in Table S4. According to the three SDBS isomers and intermediates detected, three possible degradation paths were given in Figure 9. Some studies have extensively inferred that HOMO and LUMO could theoretically determine the sites where organic pollutant molecules tend to lose or gain electrons during redox reactions [49,50]. Considering that the mass charge between the products is relatively large, and that all of them differ by 14 *m/z*, they must be the products after the attack of branched chain carbon. The above speculation could be confirmed based on the detected MS spectra at RT = 5.376–5.538 min, 3.641 min, and 3.255 min corresponding to molecular ion peaks of *m/z* = 325.2 (SDBS), 311.1, and 297.2, respectively. Of these, *m/z* = 325.2 corresponded to the anionic form of SDBS after hydrolysis in solution to release Na^+^, while *m/z* = 311.1 and 297.2 corresponded to substances where the alkane chain was decreased by one methyl and two methyl groups, respectively. According to the product types, there were three main branched forms of SDBS, including methyl, ethyl, and propyl branched chains.

According to the DFT calculation that f^−^ and f^+^ of Fukui index, the most easily oxidized sites of reactive oxygen radicals are the 1(S) bond and 16(C), 17(C), 18(C), 19(C), 20(C), and 21(C). Therefore, the sulfonyl group and branched chain carbon were not included in the final measured by-products. However, almost all of the products obtained during the reaction contain sulfonyl groups and branched carbon chains. It is possible that many intermediates were oxidized and degraded in a short time without their presence being detected. The following conclusions were obtained by deriving the degradation pathways based on the detected substances. In the presence of reactive oxygen species, SDBS was attacked in two main ways: (a) attacking the alpha-carbon, beta-carbon, or branched chain-containing carbon on the alkane chain; (b) attacking the adjacent carbon of the alkane chain and eventually forming phenolic hydroxyl groups. During the course of the reaction, signal peaks were also detected at RT = 0.9 min and 2.4 min with molecular ion peaks corresponding to *m/z* = 329 and 343 in the MS spectra. It is the further conversion product of the product after the attack of the above three branched structures, and since 329 *m/z* differs from 297 *m/z* and 343 *m/z* from 311 *m/z* by 32 *m/z*, this was the result of the obvious oxygen intervention, which is exactly in line with the (b) mode of attack of SDBS [51,52]. Among the by-products, *m/z* = 315.2 was the product of further attack on the carbon of the benzene ring of the attacked by-product of the SDBS branched chain; *m/z* = 185.0 was the product of further attack on the α carbon of the alkane chain. The degradation pathways of three SDBS isomers in VUV/O_3_ were derived from the above analysis as shown in Figure 9.

Theoretically, the site of HO•-attack could presumably appear at all C sites at the SDBS alkyl chain based on the DFT calculations, though it was more likely to occur at alpha- and beta-carbons. Nonetheless, according to the HPLC/MS, the oxidized by-products of SDBS branched carbon was detected even more frequently, and the molecular weights of the alpha- and beta-carbon cleavage products were considerably smaller than those of the basal peak cleavage by-products. At first it was the alkyl chain of SDBS that the free electrons formed by HO•-action excited the carbon atoms chemical reactivity. They are induced to react with dissolved oxygen (O_2_) and O_3_. HO• cleaved the alkyl chains at the β-position to form carbonyl groups to produced by-products (A), (B), (D), and (G). Ultimately, HO• as well as dissolved O_2_ and O_3_ transform the alkyl chains of SDBS to carboxyl groups, producing by-products (C), (E), and (H). According to the above summary, the degradation modes of the three SDBS isomers were very comparable.

### 2.5. Toxicological Analysis of Intermediates

**Figure 10:**
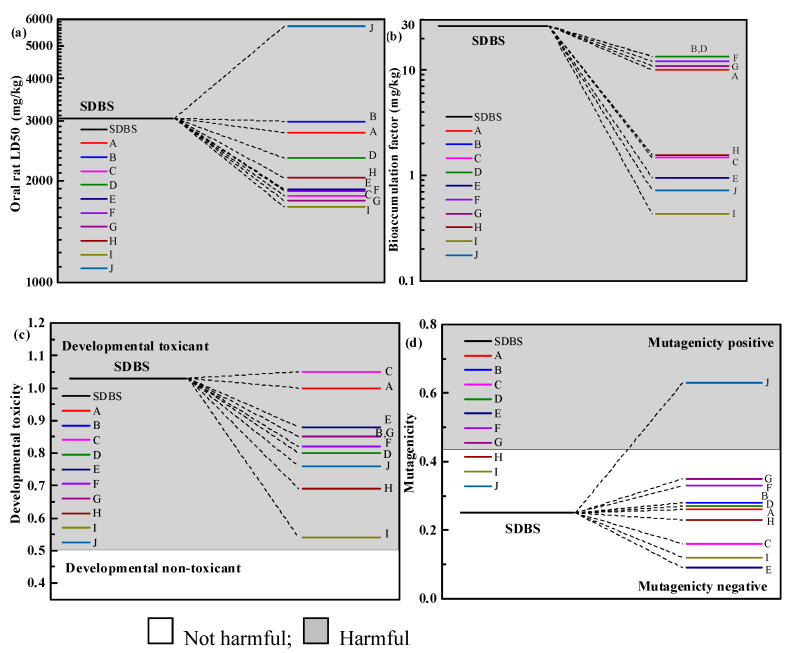
Theoretically calculated analysis of various toxicity of SDBS and its by-products: (**a**) Oral rat LD50, (**b**) bioaccumulation factor, (**c**) developmental toxicity, (**d**) mutagenicity.

Toxicological evaluations of SDBS with its degradation intermediates were performed with a toxicity assessment software tool (TEST) [53]. The structural formulae of intermediates A–J are shown in Table S4. As shown in Figure 10a, the LD50 of SDBS was estimated as 3057.19 mg/kg, which was classified to be “harmful” [6]. All by-products remained “harmful” in the process of SDBS degradation by VUV/O_3_, except for by-product J, the toxicity of other products increased. As can be seen from Figure 10b, SDBS and its intermediates/products were “harmful” in terms of bioaccumulation factors, and the bioaccumulation factors values of these compounds were reduced to different degrees compared to SDBS [54]. This indicated that the VUV/O_3_ treatment had a positive effect on the protection of the higher consumers in the organism. Figure 10c shows the developmental toxicity, which was maintained for all by-products. The developmental toxicity of all the by-products, except by-product C, were decreased. As illustrated in Figure 10d, there was an increase in the mutagenicity of 6 SDBS degradation by-products relative to the parent contaminant, but only by-product J reached a positive mutagenicity level [55]. The analysis of TEST indicated that the toxicity of most SDBS degradation by-products decreased or remained at the original level. Therefore, VUV/O_3_ treatment of SDBS has a broad application prospect on reduction of surfactants toxicity.

### 2.6. Application of VUV/O_3_ for Real Laundry Wastewater Treatment

**Figure 11:**
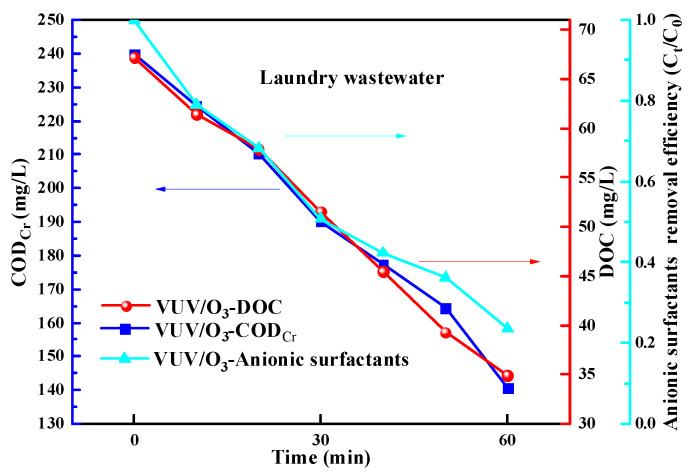
Removal efficiency of anionic surfactants (in light blue), COD_Cr_ (in dark blue) and DOC (in red) by VUV/O_3_ in laundry wastewater.

To investigate the anionic surfactant degradation effect of VUV/O_3_ in a real water matrix, we applied it to treat laundry wastewater. VUV/O_3_ could effectively remove anionic surfactants from laundry wastewater (Figure 11). The removal of surfactant was followed by the reduction of COD_Cr_ and TOC. The COD_Cr_ of laundry wastewater decreased from 239.9 to 140.6 mg/L within 60 min of VUV/O_3_ treatment. Meanwhile, the DOC was reduced from 67.22 to 34.91 mg/L under VUV/O_3_ treatment. The removal efficiency of anionic surfactants in laundry wastewater was identified to be less than that depicted above in pure water. This was probably caused by capturing of radicals and (V)UV rays by inorganic anions, protein-like and greasy contaminants existing from the treated greywater [18]. In addition, the high turbidity of the effluent may hinder the effective propagation distance of VUV and UV photons. It can be found that VUV/O_3_ was more suitable for treating low turbidity greywater after pretreatment.

## 3. Materials and Methods

### 3.1. Chemicals

The following chemicals were obtained from Aladdin (Shanghai, China): Sodium dodecylbenzene sulfonate (SDBS, AR grade, ≥99%), the radical spin-trapping agent 5,5-dimethyl-1-pyrrolineN-oxide (DMPO), chloroform (CHCl_3_), methylene blue, NaCl, Na_2_SO_4_, NaNO_3_, NaHCO_3_, tert-butanol (C_4_H_10_O, TBA), formic acid (≥98%, FA), and humic acid (HA). Ultra-pure water, required for the preparation of the reaction solution, was obtained using a Milli-Q Heal Force ultra-pure system (Millipore, Burlington, MA, USA). HCl, NaOH and H_2_SO_4_ were purchased from Beijing Chemical Works (Beijing, China). For UPLC–MS, HPLC-grade MeOH Fisher Scientific (Cranbury, NJ, USA) was used.

### 3.2. Experimental Procedures

A glass reactor with a capacity of 700 mL was used for the VUV oxidation experiments. The setup used for the tests was placed in 6 mm thick polyvinyl chloride sealed chamber, which was connected to the thermostatic water cycle (kept stable in 298 K) and also defended against UV leakage. A 6 W low-pressure mercury VUV lamp was fixed in a quartz tube to prevent water from coming into direct contact with the lamp. The low-pressure mercury lamp used (GPH150T5VH/4, Heraeus, Hanau, Germany) was mainly available in two wavelengths, 254 nm (~90%) and 185 nm (~10%), and its light intensity was tested by the degradation kinetics of photosensitizers, exhibited in Figure S1 according to existing methods [56,57]. To ensure stable output of optical power, the VUV lamps should be preheated for at least 20 min. In standard experimental procedure, a fixed amount of O_3_ (about 0.927 mg/L O_3_ dissolved in solution) was introduced into a 10 mg/L SDBS solution and simultaneously exposed to VUV for 30 min, while 10 mL samples were taken every 5 min. These samples were injected into 10 mL sampling tubes after direct filtration with a 0.45 μm microfiltration membrane.

To determine the performance of the radicals and VUV photons for the degradation of SDBS, the scavengers tert-butanol (TBA) and formic acid (FA) were added. TBA is a well-known HO• scavenger [58]; FA can absorb VUV photons in preference to H_2_O molecules (Φ_FA,185_ > Φ_H2O,185_) [59].

Several sets of univariable experiments were conducted to determine the influences of varied water constituents. The initial SDBS concentrations ([SDBS]_0_) varied from 5 to 20 mg/L. The initial solution pH was regulated from 3 to 5, 7, 9, and 11 with 0.1 M H_2_SO_4_ or 0.1 M NaOH. The influence of the presence of inorganic anions on the degradation of SDBS was measured by adding 1–2 mM Na_2_SO_4_, NaCl, NaHCO_3_, and NaNO_3_. All of the test results were the average of the three replicates.

Intermediates during SDBS degradation were identified with an Agilent 1290 Infinity/6460 LC/QQQ MS equipped with an electrospray ionization (ESI) source and operated in the negative (ESI)- electrospray ionization mode. The spray voltage (−) was 3.5 kV and capillary temperature was 300 °C. The mobile phase was a mixture of methanol and ultrapure water (0.1% formic acid) at a flow rate of 0.2 mL min^−1^. The elution process was: 0–4 min, 5–25% methanol; 4–5 min, 5–25% methanol; and 5–8 min, 25% methanol.

### 3.3. Analytical Methods

The SDBS concentration of the taken samples was measured by liquid chromatography (LC, Agilent 1260 LC, Santa Clara, CA, USA) with a C18 column. Dissolved organic carbon (DOC) was analyzed by a German Elementar TOC analyzer. The concentrations of O_3_ were determined by indigo spectrophotometry [60]. Inorganic anions were determined with a chromatograph (Metrohm, 883 Basic IC plus, Herisau, Switzerland) on a Metrosep a Supp (250.0 mm × 4.0 mm) column.

The degradation kinetics of SDBS were consistent with a pseudo-first-order kinetic formulation (Equation (11)), following our previous research [37]. *C*_0_ and *C*_t_ are initial and residual SDBS concentrations (mg/L); *k* is the degradation rate of the presented formulation (min^−1^).

−ln (*C*_t_/*C*_0_) = *k*t (11)

The spectra of radicals were recorded by electron paramagnetic resonance (EPR, JEOL JES-FA200, Akishima City, Tokyo, Japan), while all spectra were detected three times separately. O_3_ and 5,5-Dimethyl-1-pyrroline N-oxide (DMPO) were dissolved into ultrapure water and the mixture drawn to the capillary for HO• detection in EPR.

The absorbances of varied water constituents were measured using a UV-Vis spectrophotometer (UV1900, Shimadzu, Tsukuba City, Japan). The distribution ratio was calculated by Equation (12):

(12) $$P_{185\mathrm{nm},i}\%=\frac{\varepsilon_{185\mathrm{nm},i}C_{i}}{\sum_{i=1}^{i}\varepsilon_{185\mathrm{nm},i}C_{i}}\times 100$$

*P*_185nm,i_: photon absorption percentage (%) for the given solution constituent (i) at the specified 185 nm. *ε*_185nm,i_: molar absorbances (cm^−1^mol^−1^); *C*_i_: molar concentrations of the given solution constituents (mol) [34].

Density functional theory (DFT) calculations were all calculated using B3LYP/6-31+G* predictive models on Gaussian 09 software, which helped to further elucidate the radical attack on SDBS molecule in the VUV/O_3_ reaction system [61]. The molecular structural formula of generated SDBS oxidation by-products were deduced by an Agilent 1290 HPLC-MS (Santa Clara, CA, USA). Toxicological analysis of SDBS degraded intermediates were evaluated by using the Toxicity Estimation Software Tool (TEST). Use of the TEST (version 4.2.1) software is to estimate toxicity values for chemicals from their molecular structure using a variety of quantitative structure–activity relationship (QSAR) methodologies.

## 4. Conclusions

VUV/O_3_ was an effective method to degrade SDBS. The main conclusions of the present research are as follows:

(1) VUV-activated O_3_ resulted in a synergistic effect and enhanced the oxidative effect of O_3_. VUV/O_3_ could convert SDBS to inorganic more efficiently, and DOC_t_/DOC_0_ dropped to 50.37% after 30 min treatment. With VUV alone and O_3_ alone, they only reached 10.63% and 29.60%. Advanced oxidation was effective to cleavage the S-O bond of SDBS, and the final concentrations of SO_4_^2−^ increased fastest in the VUV/O_3_ process.
(2) VUV/O_3_ promoted the production of HO• compared to VUV alone and O_3_ alone, which was the major reactive species attacking SDBS molecules.
(3) The performance of VUV/O_3_ was optimal at pH 9, with lower oxidation efficiency at more acidic levels. The addition of SO_4_^2−^ hardly affected the degradation of SDBS and that of Cl^−^ and HCO_3_^−^ slightly reduced the reaction rate, except that adding NO_3_^−^ had a remarkable inhibitory effect on the process.
(4) There were three isomers in SDBS, and the degradation modes of the three SDBS isomers as three pathways were very comparable. The degradation by-products of the VUV/O_3_ process decrease in harmfulness and toxicity compared to the SDBS parent.
(5) VUV/O_3_ could effectively remove anionic surfactants from laundry wastewater. The removal efficiency of anionic surfactants in laundry wastewater was identified to be less than that depicted above in pure water.

The above results suggest that increasing the yield of hydroxyl radicals is the main goal that different processes should pursue when removing surfactants. When using the VUV/O_3_ process, a moderate increase in reaction time is recommended for deep oxidation or complete minimization of residual intermediates. This ensures that the toxic intermediates in the treated effluent are mineralized or reduced to a lower concentration.

This study contributes to the development of optimal operating conditions for the VUV/O_3_ technology and explores novel methods to remediation of surfactants.

## Data Availability

All the available data are incorporated in the manuscript.

## Supplementary Materials

The following supporting information can be downloaded at: https://www.mdpi.com/article/10.3390/molecules28083312/s1, Figure S1: Photolysis kinetics of 0.12 mM uridine (black, 254 nm) and 100 mM MeOH (purple, 185 nm) under UV or VUV irradiation; Figure S2: Natural bond orbital (NBO) analysis for the methyl isomers of SDBS molecule at B3LYP/6-31+G(d) level. (**a**) SDBS molecule structure; (**b**) The highest occupied molecular orbital (HOMO) and the lowest unoccupied molecular orbital (LUMO); (**c**) Electrostatic potential (ESP)-mapped molecular surface; Figure S3: Natural bond orbital (NBO) analysis for the ethyl isomer of SDBS molecule at B3LYP/6-31+G(d) level. (**a**) SDBS molecule structure; (**b**) The highest occupied molecular orbital (HOMO) and the lowest unoccupied molecular orbital (LUMO); (**c**) Electrostatic potential (ESP)-mapped molecular surface of SDBS; Figure S4: Natural bond orbital (NBO) analysis for the propyl isomer of SDBS molecule at B3LYP/6-31+G(d) level. (**a**) SDBS molecule structure; (**b**) The highest occupied molecular orbital (HOMO) and the lowest unoccupied molecular orbital (LUMO); (**c**) Electrostatic potential (ESP)-mapped molecular surface of SDBS; Figure S5: TIC and mass chromatography in ((a) ESI+ and (b) ESI-) mode of SDBS degradation intermediates formed during VUV/O_3_; Table S1: The methyl isomers of SDBS molecule: natural population analysis (NPA) charge populations and condensed Fukui index distribution for electrophilic attack (f^−^ and f^+^); Table S2: The ethyl isomer of SDBS molecule: natural population analysis (NPA) charge populations and condensed Fukui index distribution for electrophilic attack (f^−^ and f^+^); Table S3: The propyl isomer of SDBS molecule: natural population analysis (NPA) charge populations and condensed Fukui index distribution for electrophilic attack (f^−^ and f^+^); Table S4: Chemical formulas and main fragments (*m*/*z*) of intermediate products; Table S5: Organic characteristics and anions concentration of the laundry greywater.
