# Supplementary material for: Remediation of Surfactants Used by VUV/O3 Techniques: Degradation Efficiency, Pathway and Toxicological Analysis
Source: Molecules. 2023 Apr 8;28(8):3312. doi: 10.3390/molecules28083312 (PMC10145303; doi:10.3390/molecules28083312)
Supplement: Supplementary file 1 [file molecules-28-03312-s001.zip › molecules-2320023-supplementary.pdf]

Remediation of Surfactants Used by VUV/O_3 Techniques: Degradation Efficiency, Pathway and Toxicological Analysis

Hang Li [1], Yanling Yang [1,*], Xing Li [1] and Habib Ullah [2]

[1] College of Architecture & Civil Engineering, Faculty of Urban Construction, Beijing University of Technology, Beijing 100124, China; 17810299661@163.com (H.L.)
[2] Department of Environmental Science, Zhejiang University, Hangzhou 310058, China
* Correspondence: yangyanling@bjut.edu.cn; Tel.: +86-10-67391726

Supplementary materials include five supplementary figures and five supplementary tables.

Supplementary figures

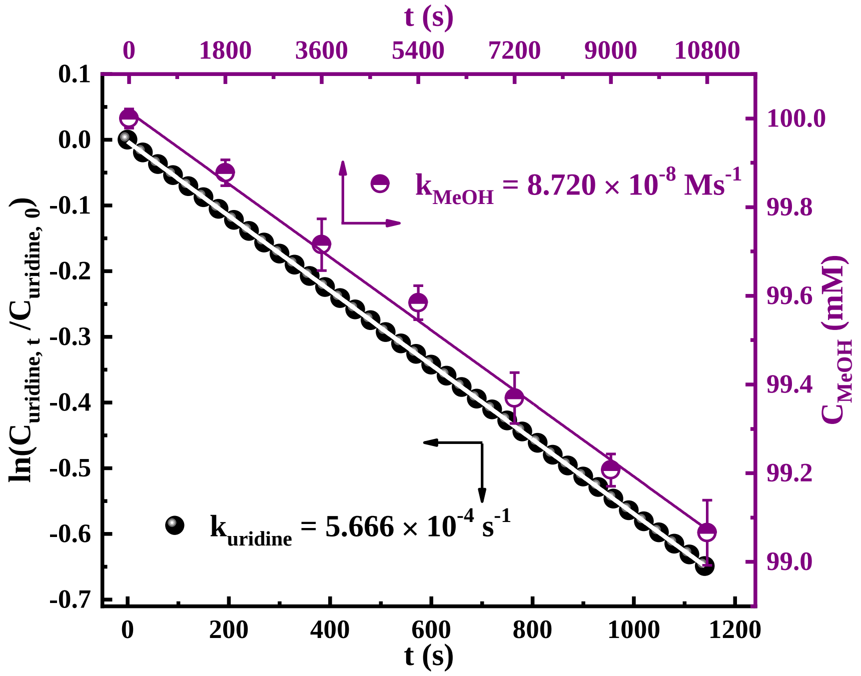

Figure S1. Photolysis kinetics of 0.12 mM uridine (black, 254 nm) and 100 mM MeOH (purple, 185 nm) under UV or VUV irradiation.

254 nm :4.98 × 10^{-4} Einstein m^{-2} s^{-1}, 185 nm: 2.17 × 10^{-5} Einstein m^{-2} s^{-1}.

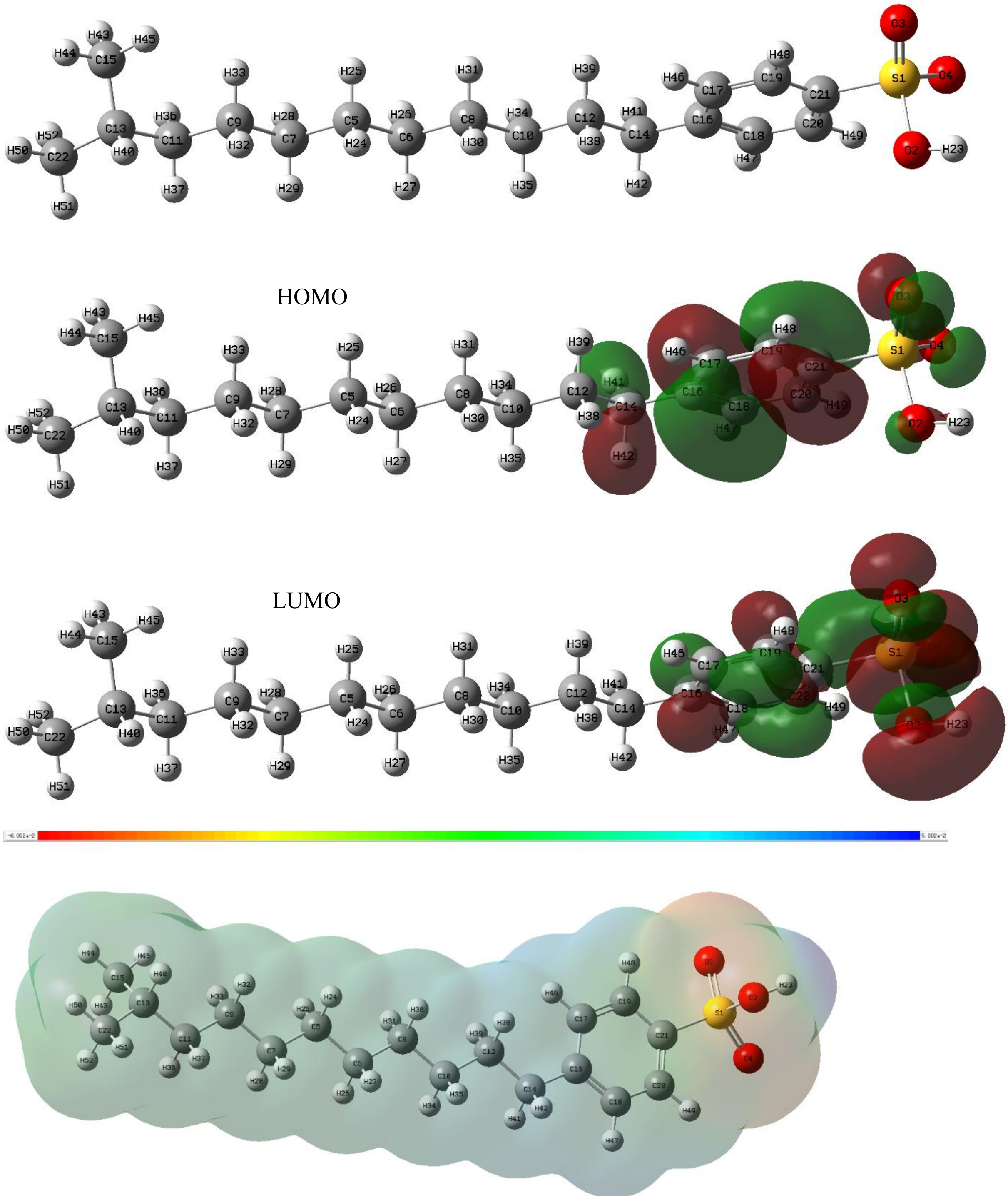

Figure S2. Natural bond orbital (NBO) analysis for the methyl isomers of SDBS molecule at B3LYP/6-31+G(d) level. (a) SDBS molecule structure; (b) The highest occupied molecular orbital (HOMO) and the lowest unoccupied molecular orbital (LUMO); (c) Electrostatic potential (ESP)-mapped molecular surface.

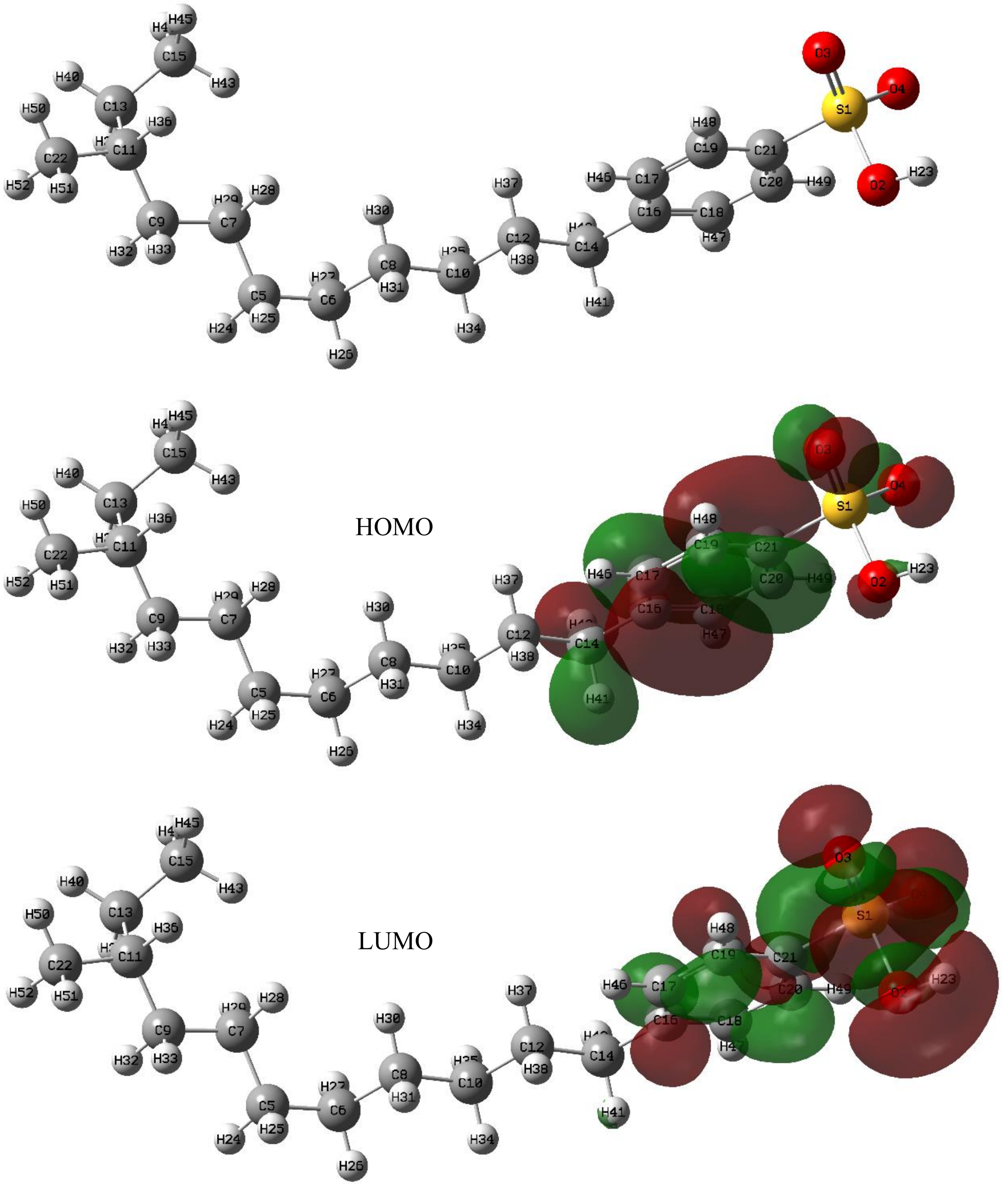
HOMO
LUMO

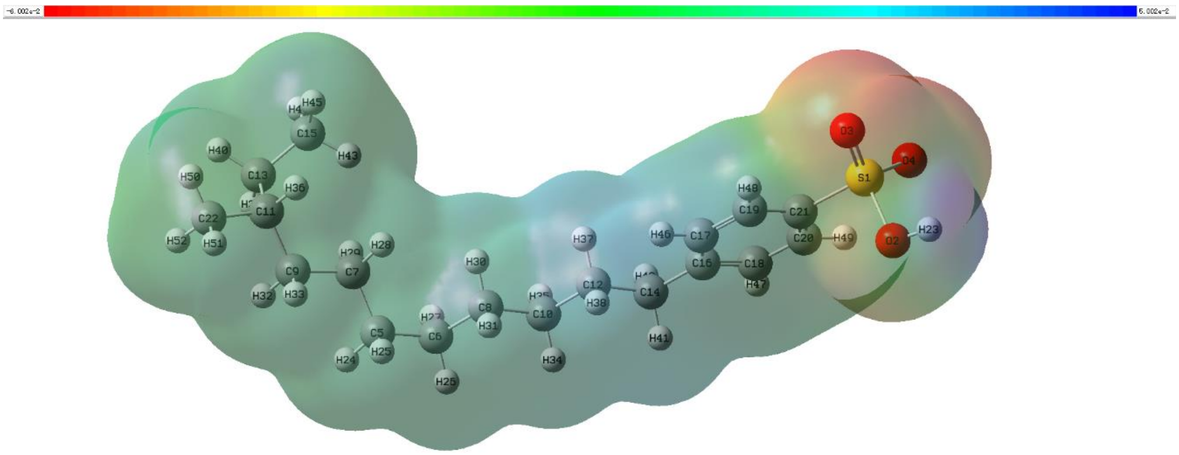

Figure S3. Natural bond orbital (NBO) analysis for the ethyl isomer of SDBS molecule at B3LYP/6-31+G(d) level. (a) SDBS molecule structure; (b) The highest occupied molecular orbital (HOMO) and the lowest unoccupied molecular orbital (LUMO); (c) Electrostatic potential (ESP)-mapped molecular surface of SDBS.

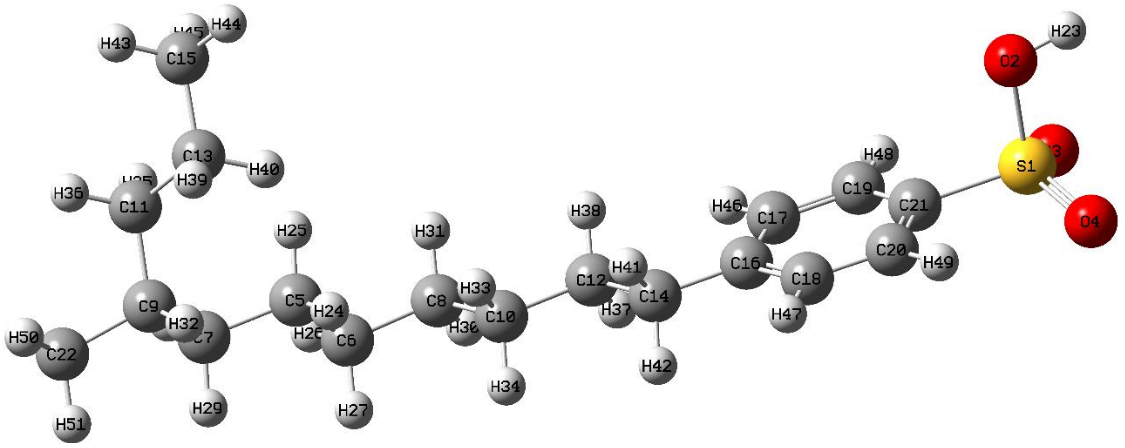

HOMO

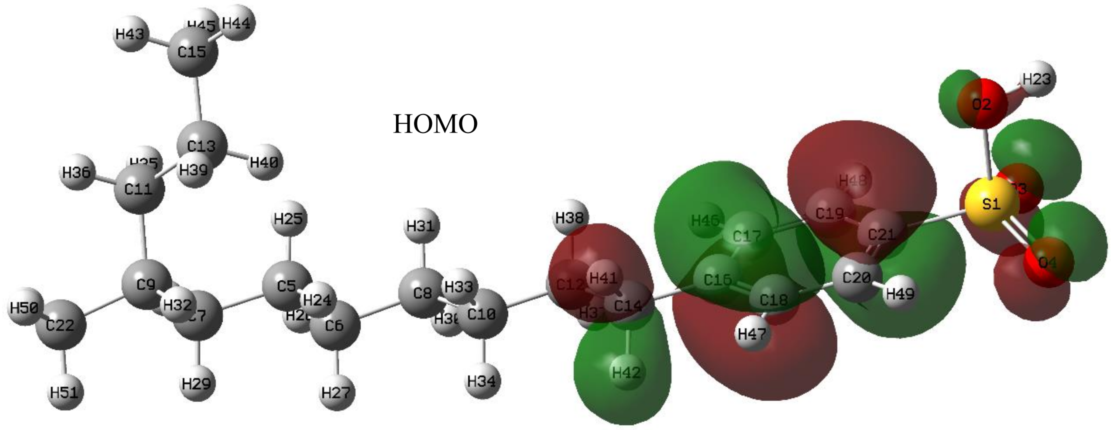

LUMO

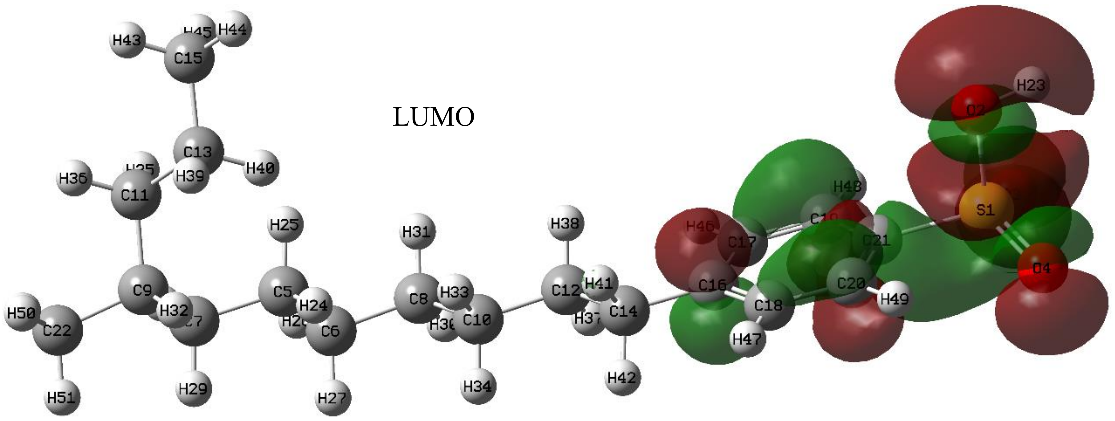

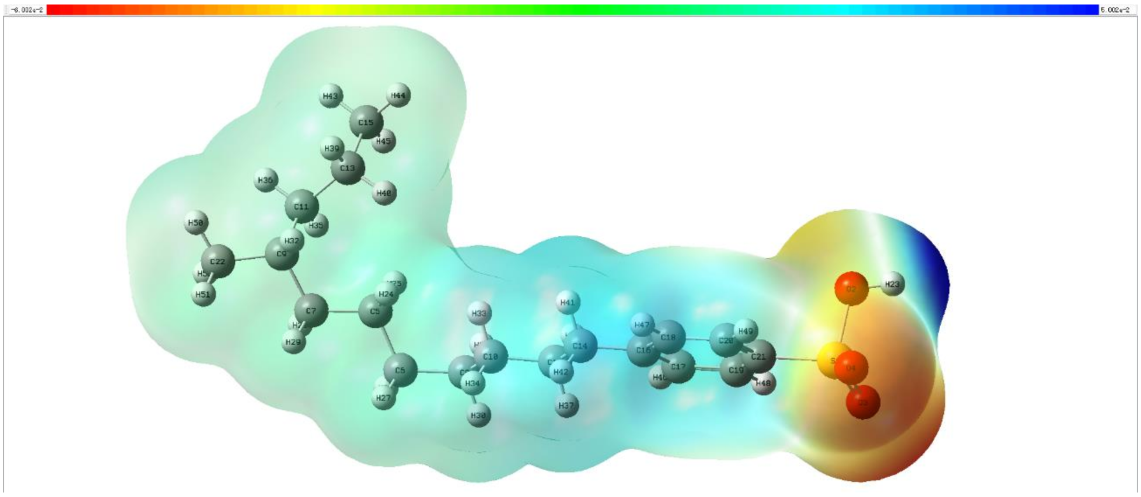

Figure S4. Natural bond orbital (NBO) analysis for the propyl isomer of SDBS molecule at B3LYP/6-31+G(d) level. (a) SDBS molecule structure; (b) The highest occupied molecular orbital (HOMO) and the lowest unoccupied molecular orbital (LUMO); (c) Electrostatic potential (ESP)-mapped molecular surface of SDBS.

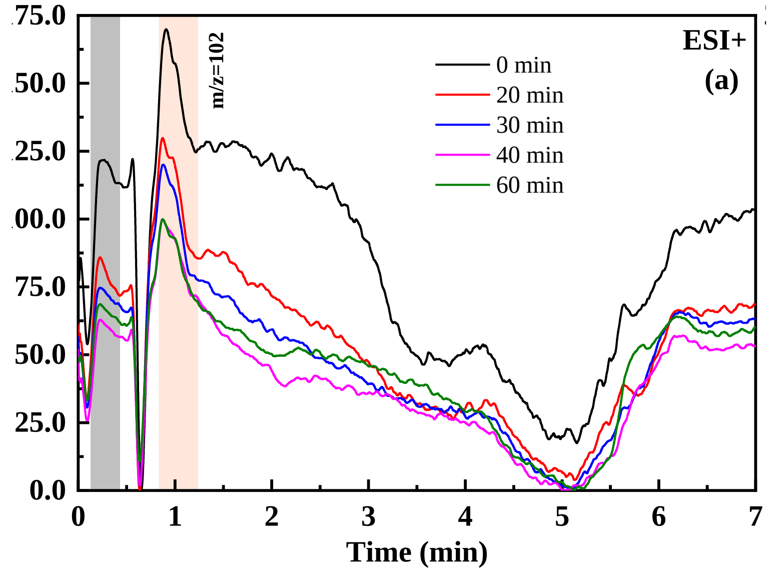

(a) ESI+

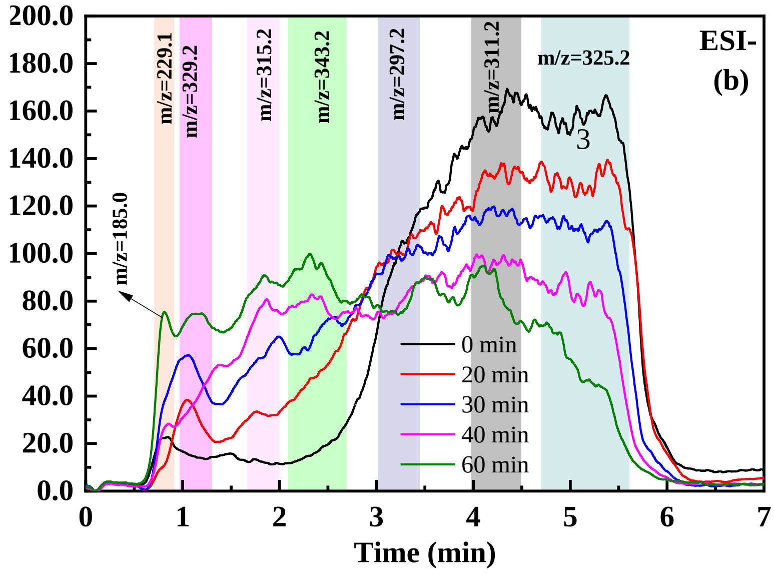

(b) ESI-

Figure S5. TIC and mass chromatography in ((a) ESI+ and (b) ESI-) mode of SDBS degradation intermediates formed during VUV/O_3.

Supplementary table

Table S1. The methyl isomers of SDBS molecule: natural population analysis (NPA) charge populations and condensed Fukui index distribution for electrophilic attack (f^- and f^+)

Atom	q(N)	q(N+1)	q(N-1)	f-	f+
1(S)	0.5223	0.4202	0.5421	0.0197	0.1022
2(O)	-0.2461	-0.3383	-0.2229	0.0232	0.0922
3(O)	-0.3227	-0.4147	-0.2695	0.0532	0.0919
4(O)	-0.3231	-0.4161	-0.2765	0.0466	0.093
5(C)	-0.0487	-0.0499	-0.0306	0.0181	0.0012
6(C)	-0.0484	-0.0503	-0.0337	0.0147	0.0019
7(C)	-0.0486	-0.0496	-0.0278	0.0208	0.001
8(C)	-0.0481	-0.0503	-0.0363	0.0118	0.0022
9(C)	-0.0493	-0.05	-0.0279	0.0214	0.0007
10(C)	-0.0468	-0.0509	-0.0376	0.0092	0.0041
11(C)	-0.0493	-0.0499	-0.0242	0.025	0.0006
12(C)	-0.0465	-0.0491	-0.0399	0.0066	0.0026
13(C)	-0.0119	-0.0122	0.0084	0.0203	0.0004
14(C)	-0.0415	-0.0551	-0.0256	0.0159	0.0136
15(C)	-0.0882	-0.0886	-0.0794	0.0088	0.0004
16(C)	0.0231	-0.056	0.0824	0.0593	0.0791
17(C)	-0.0333	-0.0766	-0.0002	0.033	0.0433
18(C)	-0.0322	-0.0801	0.0184	0.0505	0.0479
19(C)	-0.0212	-0.0792	0.0162	0.0374	0.058
20(C)	-0.0221	-0.0675	0.0048	0.0269	0.0454
21(C)	-0.0091	-0.0545	0.0486	0.0577	0.0454
22(C)	-0.0878	-0.089	-0.0671	0.0208	0.0012

Table S2. The ethyl isomer of SDBS molecule: natural population analysis (NPA) charge populations and condensed Fukui index distribution for electrophilic attack (f^{-} and f^{+})

Atom	q(N)	q(N+1)	q(N-1)	f-	f+
1(S)	0.5224	0.4202	0.544	0.0216	0.1022
2(O)	-0.2461	-0.3385	-0.2203	0.0259	0.0923
3(O)	-0.3224	-0.4144	-0.263	0.0594	0.0921
4(O)	-0.3234	-0.4163	-0.2738	0.0496	0.0929
5(C)	-0.0481	-0.0499	-0.0303	0.0178	0.0018
6(C)	-0.048	-0.0503	-0.0345	0.0136	0.0022
7(C)	-0.0498	-0.0495	-0.0341	0.0157	-0.0003
8(C)	-0.0487	-0.051	-0.044	0.0046	0.0023
9(C)	-0.049	-0.0499	-0.0253	0.0237	0.0009
10(C)	-0.0466	-0.0507	-0.0416	0.005	0.0041
11(C)	-0.0122	-0.0123	0.0085	0.0207	0.0001
12(C)	-0.0464	-0.0491	-0.0427	0.0037	0.0027
13(C)	-0.0477	-0.0484	-0.0355	0.0122	0.0007
14(C)	-0.0416	-0.0552	-0.0259	0.0156	0.0136
15(C)	-0.0883	-0.088	-0.0806	0.0078	-0.0004
16(C)	0.0231	-0.056	0.0862	0.0631	0.079
17(C)	-0.0333	-0.0767	0.0016	0.0348	0.0435
18(C)	-0.0321	-0.0798	0.0217	0.0538	0.0477
19(C)	-0.0213	-0.0788	0.0178	0.0391	0.0575
20(C)	-0.022	-0.0677	0.0057	0.0277	0.0457
21(C)	-0.009	-0.0544	0.0513	0.0603	0.0454
22(C)	-0.0877	-0.0892	-0.071	0.0168	0.0015

Table S3. The propyl isomer of SDBS molecule: natural population analysis (NPA) charge populations and condensed Fukui index distribution for electrophilic attack (f^- and f^+)

Atom	q(N)	q(N+1)	q(N-1)	f-	f+
1(S	0.5224	0.4203	0.5441	0.0217	0.1021
2(O)	-0.2461	-0.3382	-0.2207	0.0253	0.0921
3(O)	-0.3228	-0.4147	-0.2629	0.0599	0.0919
4(O	-0.3229	-0.4158	-0.2729	0.05	0.0929
5(C)	-0.0498	-0.0497	-0.0378	0.012	-0.0001
6(C)	-0.0477	-0.0501	-0.0323	0.0155	0.0024
7(C)	-0.0489	-0.0502	-0.026	0.0229	0.0012
8(C)	-0.0477	-0.0502	-0.0365	0.0111	0.0025
9(C)	-0.0118	-0.0121	0.0083	0.0201	0.0003
10(C)	-0.0474	-0.0516	-0.0427	0.0047	0.0042
11(C)	-0.0493	-0.0498	-0.0353	0.014	0.0005
12(C)	-0.0463	-0.0488	-0.0419	0.0044	0.0025
13(C)	-0.0491	-0.0484	-0.0409	0.0082	-0.0007
14(C)	-0.0416	-0.0552	-0.0256	0.016	0.0137
15(C)	-0.0875	-0.0879	-0.0784	0.0091	0.0004
16(C)	0.023	-0.0561	0.0875	0.0645	0.0791
17(C)	-0.0333	-0.0765	0.0032	0.0365	0.0432
18(C)	-0.0322	-0.0803	0.0224	0.0545	0.0481
19(C)	-0.0212	-0.0793	0.0192	0.0404	0.0581
20(C)	-0.0222	-0.0675	0.0063	0.0285	0.0453
21(C)	-0.0091	-0.0545	0.0533	0.0624	0.0454
22(C)	-0.0876	-0.0895	-0.0753	0.0123	0.0019

Table S4. Chemical formulas and main fragments (*m*/*z*) of intermediate products

Intermediate products ID	*m*/*z*	proposed structure
A	311.1(-)	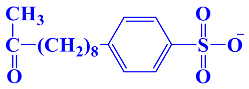
B	325.2(-)	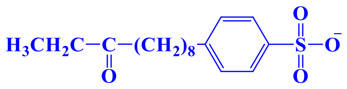
C	329.0(-)	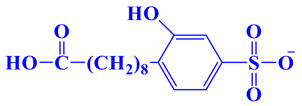
D	325.2(+)	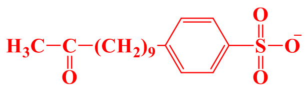
E	343(-)	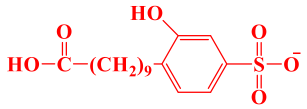
F	325.2(-)	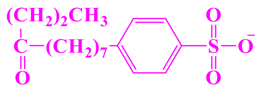
G	297.2(-)	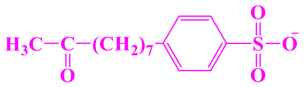
H	315(-)	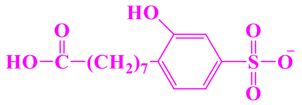
I	185(-)	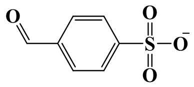
J	102(+)	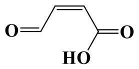

Table S5. Organic characteristics and anions concentration of the laundry wastewater

Water samples	Water sample characteristics			
	UV_{254}	COD_{Cr} (mg/L)	DOC (mg/L)	pH
laundry wastewater	0.058 ± 0.001	239.9	67.22± 0.132	7.67 ± 0.13

Water samples	Anions(mg/L)				
	Anionic surfactants	Cl^-	NO_3^-	PO_4^{2-}	SO_4^{2-}
laundry wastewater	14.48	79.87	8.19	1.96	107.81

Real laundry wastewater was taken from a university laundry room in the Chaoyang district, Beijing. The greywater from a single washing machine of twice washes was mixed and filled into a 10 L bucket.
